# Immune Centroids Oversampling Method for Binary Classification

**DOI:** 10.1155/2015/109806

**Published:** 2015-03-05

**Authors:** Xusheng Ai, Jian Wu, Victor S. Sheng, Pengpeng Zhao, Zhiming Cui

**Affiliations:** ^1^The Institute of Information Processing and Application, Soochow University, Suzhou 215006, China; ^2^Department of Computer Science, University of Central Arkansas, Conway, AR 72035, USA

## Abstract

To improve the classification performance of imbalanced learning, a novel oversampling method, immune centroids oversampling technique (ICOTE) based on an immune network, is proposed. ICOTE generates a set of immune centroids to broaden the decision regions of the minority class space. The representative immune centroids are regarded as synthetic examples in order to resolve the imbalance problem. We utilize an artificial immune network to generate synthetic examples on clusters with high data densities, which can address the problem of synthetic minority oversampling technique (SMOTE), which lacks reflection on groups of training examples. Meanwhile, we further improve the performance of ICOTE via integrating ENN with ICOTE, that is, ICOTE + ENN. ENN disposes the majority class examples that invade the minority class space, so ICOTE + ENN favors the separation of both classes. Our comprehensive experimental results show that two proposed oversampling methods can achieve better performance than the renowned resampling methods.

## 1. Introduction

The class imbalance problem typically occurs when, in a binary classification problem, there are more training examples of one class than those of the other class. This situation is known as the class imbalance problem [1]. Under the circumstances, most standard algorithms fail to properly represent the distributive characteristics of complex imbalanced datasets and then provide unfavorable accuracies across examples of two classes [2]. Furthermore, it is worth pointing out that the minority class is usually the one that has the highest interest from a learning point of view and it also implies a great cost when it is not well classified [3].

Standard classification learning algorithms are often biased towards the majority class (known as the negative class). Therefore, it is not unusual that there is a higher misclassification rate for the minority class (i.e., the positive class) instances. In order to deal with this problem, a large number of approaches have been proposed to counter the sparsity in the distribution. Among them, the “synthetic minority oversampling technique” (SMOTE) [4] has become one of the most renowned approaches in this area. It can be essential to provide new related information on the positive class to the learning algorithm, in addition to undersampling majority class, which is completely different from undersampling majority class. Batista et al. proposed an integration method called SMOTE + ENN [5], which uses Wilson's edited nearest neighbor (denoted as ENN) rule [6] to remove examples whose classes differ from the classes of at least a half of their nearest neighbors. Han et al. present two new minority oversampling methods, borderline-SMOTE1 and borderline-SMOTE2 [7], in which only the minority class examples near the borderline are oversampled. Later, Bunkhumpornpat et al. published safe-level-SMOTE [8]. Their approach samples minority instances along the safe level that is computed using nearest neighboring minority instances. Ramentol et al. came up with another oversampling method with the application of an editing technique based on the rough set theory and the lower approximation of a subset [9].

In this paper we present an immune centroids oversampling technique (ICOTE) based on immune network theory. We utilize an aiNet model [10] to generate immune centroids of clusters of high data density. Our work resamples the minority class by introducing immune centroids of clusters of minority class examples. The resampling creates larger but less specific decision regions. Meanwhile, we also integrate our ICOTE with ENN together, that is, ICOTE + ENN. ICOTE + ENN can not only sample minority class examples but also dispose majority class examples that invade the minority class space. We expect that this hybrid method excels ICOTE in terms of the separation of both classes. Our experimental results show that both ICOTE and ICOTE + ENN achieve better performance in the application of three paradigms, comparing with the existing methods.

The rest of this paper is organized as follows. We review related work in Section 2.  Section 3 presents our proposed oversampling methods ICOTE and ICOTE + ENN. Our experimental results and comparisons are shown in Section 4. Finally, we conclude this paper in Section 5.

## 2. Related Work

In order to deal with imbalanced issues, some articles studied different resampling techniques, which change the class distribution. These articles empirically showed that applying a preprocessing step in order to balance the class distribution usually is a useful solution [5, 11–13]. Furthermore, the main advantage of these techniques is that they are independent of the underlying classifier. Resampling techniques can be categorized into three groups or families:

1. undersampling methods, which create a subset of the original dataset by eliminating some instances (usually majority class instances),
2. oversampling methods, which create a superset of the original dataset by replicating some instances or creating new instances from the existing ones,
3. hybrids methods, which combine both sampling approaches from the above.

López et al. [14] evaluated various sampling methodologies on a variety of datasets with different class distributions. They selected a collection of methods belonging to the three categories. They concluded that both SMOTE [4] and SMOTE + ENN [5] are more applicable and give very good results for datasets with various imbalanced rates. They also noted that the sophisticated sampling techniques did not give any clear advantage in the domains considered.

**Figure 1:**
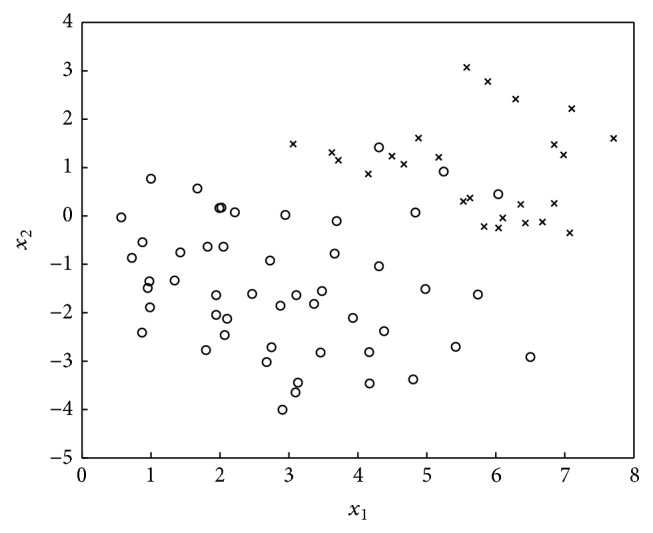
Data sample composed of 50 majority class examples and 25 minority class examples.

**Figure 2:**
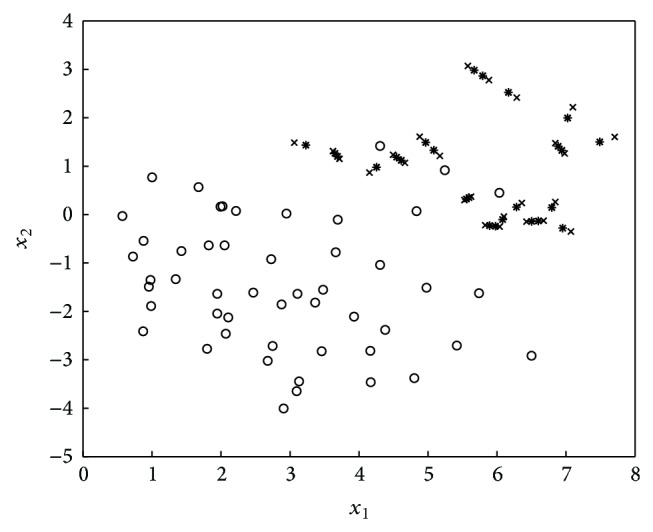
SMOTE introduces each synthetic example (in star) along the line segment joining the 2 minority class neighbors.

The SMOTE algorithm [4] oversamples the minority class. Specifically, it introduces synthetic examples along the line segments through joining some/all of the *k* minority class nearest neighbors. Depending on the amount of oversampling required, neighbors from the *k*-nearest neighbors are randomly chosen. Figures 1-2 illustrate the distribution change in the application of SMOTE [4].

**Figure 3:**
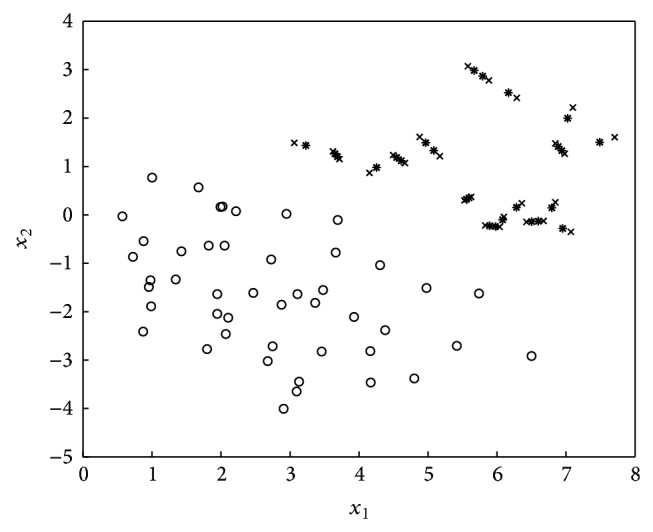
SMOTE introduces synthetic examples (in star) along the line segments via joining 2 minority class neighbors and ENN disposes the majority class examples in the minority class space.

SMOTE + ENN [5] uses ENN to remove examples from both classes. Since some majority class examples might invade the minority class space and vice versa, SMOTE + ENN [5] reduces the possibility of overfitting introduced by synthetic examples. The cleaning result of ENN is illustrated in Figure 3.

Jo and Japkowicz [15] discussed whether class imbalance is truly responsible for this degradation or whether it can be explained in some other ways. Their experiments suggest that the problem is not directly caused by class imbalance, but rather that class imbalances may yield small groups which, in turn, will cause this degradation. SMOTE [4] and its successors enrich the minority class space without considering data intrinsic characteristics such as small groups. The SMOTE-based methods might create the synthetic examples which underrepresent actual clusters or are attributed to noisy data. We will describe how our method overcomes the inherent drawback in the subsequent section.

## 3. Our Methods

In this section, we first briefly introduce the basic concepts and knowledge of immune systems. After that, we present our oversampling method ICOTE based on immune network theory and its improved version ICOTE + ENN.

### 3.1. Immune Systems

Before discussing our method, we sketch a few aspects of the human adaptive immune system. The immune systems guard our bodies against infections due to the attacks of antigens. The surface receptors on B-cells (one kind of lymphocyte) are able to recognize specific antigens. The response of a receptor to an antigen can activate its hosting B-cell. Activated B-cell then proliferates and differentiates into memory cells. Memory cells secrete antibodies to neutralize the pathogens through complementary pattern matching. During the proliferation of the activated B-cells, a mutation mechanism is employed to create diverse antibodies by altering the gene segments. Some of the mutants may be a better match for the corresponding antigen. In order to be protective, the immune system must learn to distinguish between our own (self) cells and malefic external (nonself) invaders. This process is called self/nonself discrimination: those cells recognized as self do not promote an immune response, and the system is said to be tolerant to them, while those that are not provoke a reaction resulting in their elimination.

The immune network theory, as originally proposed in [16], hypothesizes a novel viewpoint of lymphocyte activities, natural antibody production, preimmune repertoire selection, tolerance and self/nonself discrimination, memory, and the evolution of the immune system. It was suggested that the immune system is composed of a regulated network of cells and molecules that recognize one another. The immune cells can respond either positively or negatively to the recognition signal (antigen or another immune cell or molecule). A positive response would result in cell proliferation, cell activation, and antibody secretion, while a negative response would lead to tolerance and suppression.

Learning in the immune system involves raising the population size and affinity of those lymphocytes that have proven themselves valuable by having recognized any antigen. Burnet [17] introduced clonal selection theory by modifying Jerne's theory. The theory stated that, in a preexisting group of lymphocytes (specifically B-cells), a specific antigen only activates (i.e., selection) its counter-specific cell so that a particular cell is induced to multiply (producing its clones) for antibody production. With repeated exposures to the same antigen, the immune system produces antibodies of successively greater affinities. A secondary response elicits antibodies with greater affinity than in a primary response. Based on the clonal selection principle, de Castro and von Zuben [18] proposed a computational implementation of the clonal selection principle that explicitly takes into account the affinity maturation of the immune response. He also defined aiNet (an artificial immune network model) for data analysis [10]. The aiNet is an edge-weighted graph, not necessarily fully connected, composed of a set of nodes, called antibodies, and sets of node pairs called edges with an assigned number called weight, or connection strength, associated with each connected edge. The aiNet clusters serve as internal images (mirrors) responsible for mapping the existing clusters in the dataset into network clusters. These clusters map those of the original dataset. The shape of the spatial distribution of antibodies follows the shape of the antigenic spatial distribution.

### 3.2. Immune Centroids Resampling

In this paper we present a resampling method based on immune network theory. We use the aiNet model [10] to generate antibody-derived synthetic examples and extend a training set to balance sample distribution. The immune synthetic examples represent internal images of original minority class examples, so we call the resampling method immune centroids oversampling technique (ICOTE).

Before explaining ICOTE, we introduce some notations to describe the resampling method. Given a set of *n* labeled examples $D_{n}=\{\left(\vec{X_{1}},Y_{1}\right),\ldots ,\left(\vec{X_{n}},Y_{n}\right)\}$ with input vectors $\vec{X_{i}}\in R^{d}$ and class label *Y*_*i*_ ∈ {negative, positive}, we measure the affinity (complementarity level) of the antigen-antibody match using Euclidean distance. As we know, the Euclidean distance of two vectors is

(1) $$\begin{array}{c} \mathrm{dist}\left(\vec{X_{i}},\vec{X_{j}}\right)=\sqrt{\sum {\left(X_{i}^{m}-X_{j}^{m}\right)}^{2}}, \end{array}$$

where *m* is the dimension of each vector. The antigen-antibody affinity is inversely proportional to the Euclidean distance. The smaller the distance, the higher the affinity, and vice versa.

Our ICOTE includes four major steps as follows.

#### 3.2.1. Attribute Selection

In order to reduce computational cost, we first remove the attributes whose values are constant:

(2) fselect(X→)=X1,…,Xk,Xk+l+1,…,XdT,Xk+1≡C1,…,Xk+l=Cl, Ci∈R.

#### 3.2.2. Unit-Based Normalization

Then we adjust the values of attributes on different scales to a notionally common scale [0, 1]:

(3) $$\begin{array}{c} \mathrm{norm}\left(x\right)=\frac{x-x_{\min}}{x_{\max}-x_{\min}}. \end{array}$$

#### 3.2.3. Immune Centroids Generation

There are three steps for generating immune centroids. First, the selected antibodies $\vec{Ab}$ are going to proliferate (clone) proportionally to their antigenic affinity: the higher the affinity, the larger the clone size *nc* for each selected antibody:

(4) $$\begin{array}{c} \text{clone}\left(\vec{Ab}\right)=\left\{\vec{Ab_{1}},\ldots ,\vec{Ab_{nc}}\right\}. \end{array}$$

Next, each antibody $\vec{Ab}$ from the clone set will suffer a mutation with a rate *α*_*k*_, which is inversely proportional to the antigenic affinity of its parent antibody $\vec{Ag}$:

(5) mutateAb→=Ab→+αkAb→−Ag→,αk=1d∗dist⁡Ab→,Ag→.

And then we eliminate the memory antibodies (denoted as *M*) with a low antigen-antibody affinity (clonal suppression) *f*_*ij*_ and a high antibody-antibody affinity (network suppression) *f*_*ij*_′:

(6) $$\begin{array}{c} \text{suppress}\left(M\right)=M-M_{f_{ij}>T_{1}}-M_{f_{ij}^{\prime }>T_{2}},\;T_{1}\in R,\;\;T_{2}\in R. \end{array}$$

#### 3.2.4. Denormalization

Next, we denormalize memory antibodies *M* and make synthetic examples identical to sample distribution:

(7) $$\begin{array}{c} \text{de-norm}\left(x\right)=x_{\min}+\left(x_{\max}-x_{\min}\right)x. \end{array}$$

#### 3.2.5. Attribute Replacement

At the end, we put back constant-value attributes:

(8) de-fselect(S→)=S1,…,Sk,C1,…,Cl,Xk+l+1,…,XdT,Ci∈R.

Correspondingly, the algorithm is described as shown in Algorithm 1.

**Figure 4:**
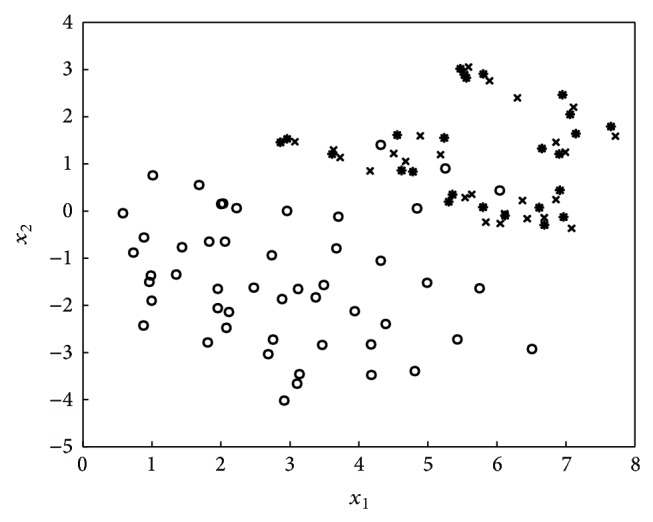
ICOTE introduces the immune centroids (in star) following the shape of the neighboring minority class examples.

ICOTE samples minority class examples to generate memory antibodies (immune centroids). The shape of the spatial distribution of the immune centroids follows that of the minority class examples. Therefore, it avoids more small groups or outliers introduced by oversampling. For instance, we depict the immune centroids in Figure 4. Intuitively, each of them in star shares the same group with one or several neighboring minority class examples. Introducing immune centroids for learning not only creates larger and less specific decision regions but also decreases the likelihood of overfitting occurring, which is the major drawback of SMOTE [4].

**Figure 5:**
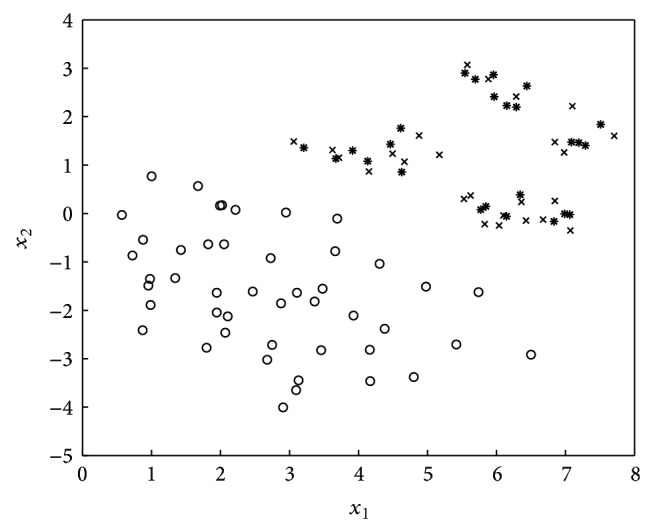
ICOTE introduces the immune centroids (in star) following the shape of the neighboring minority class examples and ENN [6] disposes the majority class examples in the minority class space.

We also propose an integrated method called ICOTE + ENN, integrating ICOTE with the Wilson's edited nearest neighbor rule (i.e., ENN) [6]. In this integrated method, ICOTE oversamples minority class examples, and ENN discards “dirty” examples deviating from the majority class space. When the class of an example differs from the class of more than a half of the nearest neighbors, the example will be removed from the training set. The result of the integrated method is illustrated in Figure 5. Figure 5 shows that the hybrid method makes the two class spaces separated. In the next section, we will show our empirical results for our two methods.

## 4. Experiments

In this section, we will investigate the performance of our proposed oversampling methods ICOTE and ICOTE + ICOTE and compare them with the existing well-known oversampling methods.

### 4.1. Experimental Settings

Our experiments are conducted based on three base classifiers: *k*NN, C4.5, and SVM. We use these algorithms, since they are available within the KEEL software tool [19]. In the experiments, the parameter values are set based on the recommendations from the corresponding authors. The specific settings are as follows.

1. Instance based learning (*k*NN) [20]: in this algorithm, we set *k* = 1 and use the Euclidean distance metric.
2. C4.5 decision tree [21]: for C4.5, we set a confidence level as 0.25 and the minimum number of item sets per leaf as 2 and use pruning.
3. Support vector machines (SVM) [22]: for SVM, we choose Polykernel reference functions, setting an internal parameter 1.0 for the exponent of each kernel function and a penalty parameter of the error term as 1.0.

We conduct experiments on 38 datasets from the KEEL dataset repository [23], whose characteristics are summarized in Table 1, namely, the number of examples (#Ex.), number of attributes (#Atts.), and instance ratio (IR). The experiments are evaluated in terms of one of the popular metrics, the area under the ROC curve (AUC) [24, 25]. The experimental results are obtained based on 5-fold cross-validation. We choose 5-fold cross-validation, because it can keep sufficient positive class instances in different folds. Thus, we can avoid additional problems in the data distribution which were discussed in [26, 27], especially for highly imbalanced datasets.

We must point out that the dataset partitions employed in this paper are available from the KEEL dataset repository [23], so that researchers can use the same data partitions for comparisons.

### 4.2. Evaluation in Imbalanced Domains

In imbalanced domains, a well-known approach to unify these measures and to produce an evaluation criterion is to use the receiver operating characteristic (ROC) graphic [24]. This graphic allows the visualization of the trade-off between the benefits (TP_rate_) and costs (FP_rate_), as it evidences that any classifier cannot increase the number of true positives without also increasing the false positives. The area under the ROC curve (AUC) [25] corresponds to the probability of correctly identifying which one of the two stimuli is noise and which one is signal plus noise. The AUC provides a single measure of a classifier's performance for evaluating which model is better on average. The AUC measure is computed just by obtaining the area of the graphic:

(9) $$\begin{array}{c} \text{AUC}=\frac{1+\text{T}{\text{P}}_{\text{rate}}-\text{F}{\text{P}}_{\text{rate}}}{2}. \end{array}$$

AUC combines the individual measures of both the positive and negative classes so that we can utilize it to measure quality results of different paradigms for imbalanced data.

### 4.3. Experimental Results

In this section, we investigate the performance of the resampling methods on the imbalanced datasets listed in Table 1.

As shown in the previous work [14, Table 4] on the keel datasets, SMOTE [4] and SMOTE + ENN [5] have the highest rank for the three classification algorithms (*k*NN, C4.5, and SVM) used in their study, and both ADASYS [28] and SL-SMOTE [29] achieve the 2nd highest AUC values. So we select these four resampling algorithms and compare our ICOTE and ICOTE + ENN with them. The average AUC results of different resampling methods with three base learners *k*NN, C4.5, and SVM over all 38 datasets are shown in Table 2. Besides, we also have the experimental results obtained based on the three base learners directly without using resampling techniques, which is denoted as “none” in Table 2. Please note that our experimental results on each dataset are shown in the Appendix.

From Table 2, we can see that our methods ICOTE and ICOTE + ENN perform much better than the other four resampling methods, on all three base learners. And ICOTE + ENN does improve the performance of ICOTE. Our experimental results also show that SMOTE and SMOTE + ENN perform better than SL-SMOTE and ADASYN. SMOTE + ENN does improve the performance of SMOTE a little on all the three base learners. Between SL-SMOTE and ADASYN, SL-SMOTE performs better. That is, ADASYN is the worst among the six resampling methods.

**Figure 6:**
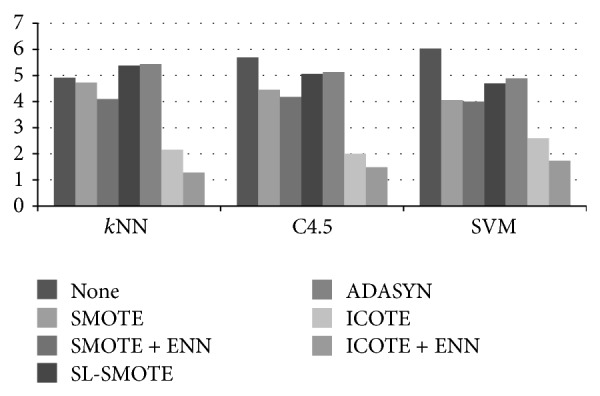
The average ranks of the resampling methods under three base learners, respectively.

Besides the average results shown in Table 2, we also rank the resampling methods on each dataset with each base learner. The average ranks of each method with each base learner are shown in Figure 6. From Figure 6, we can see that the average rank of ICOTE + ENN is the best under any one of the three base learners. ICOTE ranks the second consistently. SMOTE + ENN always ranks the third. SMOTE always ranks the fourth. It is obvious that “none” (without using resampling techniques) performs the worst when either C4.5 or SVM is used as the base learner. Between SL-SMOTE and ADASYN, SL-SMOTE always performs better than ADASYN. These conclusions are consistent with the conclusions we made based on the average AUC, shown in Table 2.

For the sake of finding out which algorithms are distinctive among the pair comparisons of these methods, we carry out a Shaffer post hoc test [30], which is shown in Tables 3–5. In these tables, a “+” symbol implies that the algorithm in the row is statistically better than the one in the column, “−” implies the contrary, and “=” means that the two algorithms compared show no significant difference. In brackets, the unadjusted *P* value associated with each comparison is also presented. Shaffer's procedure rejects those hypotheses that have an unadjusted *P* value ≤0.002.

In order to explain why ICOTE and ICOTE + ENN obtain the highest performance, we may emphasize two feasible reasons. The first one is related to the addition of significant information within the minority class examples by including immune centroids of clusters. These immune centroids allow the formation of larger clusters to help the classifiers to separate both classes, and its cleaning procedure also adds benefits to the generalization ability during learning. The second reason is that the immune centroids represent inherent clusters of the minority class examples and overcome the limitation that synthetic examples form new clusters or outliers.

## 5. Conclusions

In this paper we present two overresampling methods based on immune network theory. We draw the conclusions based on our experimental results and analyses as follows.

1. ICOTE samples minority class examples to generate immune centroids as synthetic examples, which is far different from renowned resampling methods without considering sample architecture.
2. ICOTE introduces minority class examples and ENN disposes majority class examples in the minority data space. ICOTE + ENN favors separating both classes.
3. We compare our proposed methods ICOTE and ICOTE + ENN with representative resampling methods. Our experimental results showed that our approaches make significant improvement.

## Figures and Tables

**Algorithm 1:**
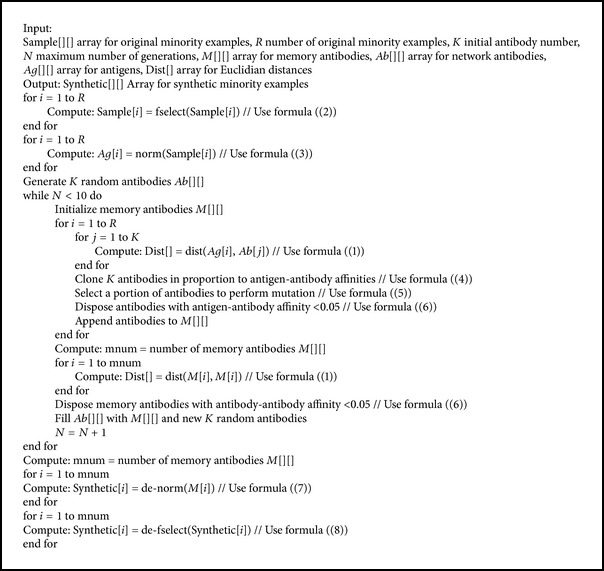


**Table 1:** The characteristics of imbalanced datasets.

Name	#Ex.	#Attrs	IR	Name	#Ex.	#Attrs	IR
glass1	214	9	1.82	yeast-2_vs_4	514	8	9.08
ecoli-0_vs_1	220	7	1.86	yeast-0-5-6-7-9_vs_4	528	8	9.35
wisconsinimb	683	9	1.86	vowel0	988	13	10.1
pimaimb	768	8	1.9	glass-0-1-6_vs_2	192	9	10.29
iris0	150	4	2	glass2	214	9	10.39
glass0	214	9	2.06	shuttle-c0-vs-c4	1829	9	13.87
yeast1	1484	8	2.46	yeast-1_vs_7	459	8	13.87
habermanimb	306	3	2.68	glass4	214	9	15.47
vehicle2	846	18	2.52	ecoli4	336	7	13.84
vehicle1	846	18	2.52	glass-0-1-6_vs_5	184	9	19.44
vehicle3	846	18	2.52	shuttle-c2-vs-c4	129	9	20.5
glass-0-1-2-3_vs_4-5-6	214	9	3.19	yeast-1-4-5-8_vs_7	693	8	22.1
vehicle0	846	18	3.23	glass5	214	9	22.81
ecoli1	336	7	3.36	yeast-2_vs_8	482	8	23.1
newthyroid2	215	5	4.92	yeast4	1484	8	28.41
new-thyroid1	215	5	5.14	yeast-1-2-8-9_vs_7	947	8	30.56
ecoli2	336	7	5.46	yeast5	1484	8	32.78
glass6	214	9	6.38	ecoli-0-1-3-7_vs_2-6	281	7	39.15
yeast3	1484	8	8.11	yeast6	1484	8	39.15

**Table 2:** Average AUC results of different resampling methods with *k*NN, C4.5, and SVM as the base learner, respectively.

	*k*NN	C45	SVM
None	0.791	0.793	0.712
SMOTE	0.839	0.839	0.850
SMOTE + ENN	0.848	0.840	0.850
SL-SMOTE	0.836	0.828	0.844
ADASYN	0.804	0.819	0.843
ICOTE	**0.955**	**0.936**	**0.871**
ICOTE + ENN	**0.969**	**0.947**	**0.887**

**Table 3:** The Shaffer test for the oversampling techniques with *k*NN using the AUC measure.

	None	SMOTE	SMOTE + ENN	SL-SMOTE	ADASYN	ICOTE	ICOTE + ENN
None	X	−(0.001)	=(0.000)	=(0.009)	=(0.957)	−(0.000)	−(0.000)
SMOTE	+(0.001)	X	=(0.633)	=(0.441)	+(0.001)	−(0.000)	−(0.000)
SMOTE + ENN	=(0.000)	=(0.633)	X	=(0.212)	+(0.000)	−(0.000)	−(0.000)
SL-SMOTE	=(0.009)	=(0.441)	=(0.212)	X	=(0.010)	−(0.000)	−(0.000)
ADASYN	=(0.957)	−(0.001)	−(0.000)	=(0.010)	X	−(0.000)	−(0.000)
ICOTE	+(0.000)	+(0.000)	+(0.000)	+(0.000)	+(0.000)	X	=(0.167)
ICOTE + ENN	+(0.000)	+(0.000)	+(0.000)	+(0.000)	+(0.000)	=(0.167)	X

**Table 4:** The Shaffer test for the oversampling techniques with C4.5 using the AUC measure.

	None	SMOTE	SMOTE + ENN	SL-SMOTE	ADASYN	ICOTE	ICOTE + ENN
None	X	−(0.00)	−(0.000)	=(0.117)	=(0.193)	−(0.000)	−(0.000)
SMOTE	+(0.001)	X	=(0.541)	=(0.300)	=(0.193)	−(0.000)	−(0.000)
SMOTE + ENN	+(0.000)	=(0.560)	X	=(0.009)	=(0.056)	−(0.000)	−(0.000)
SL-SMOTE	=(0.117)	=(0.300)	=(0.009)	X	=(0.790)	−(0.000)	−(0.000)
ADASYN	=(0.193)	=(0.193)	=(0.056)	=(0.790)	X	−(0.000)	−(0.000)
ICOTE	+(0.000)	+(0.000)	+(0.000)	+(0.000)	+(0.000)	X	=(0.353)
ICOTE + ENN	+(0.000)	+(0.000)	+(0.000)	+(0.000)	+(0.000)	=(0.353)	X

**Table 5:** The Shaffer test for the oversampling techniques with SVM using the AUC measure.

	None	SMOTE	SMOTE + ENN	SL-SMOTE	ADASYN	ICOTE	ICOTE + ENN
None	X	−(0.000)	−(0.000)	=(0.005)	=(0.009)	−(0.000)	−(0.000)
SMOTE	+(0.000)	X	=(0.710)	=(0.352)	=(0.254)	−(0.001)	−(0.000)
SMOTE + ENN	+(0.000)	=(0.710)	X	=(0.193)	=(0.130)	=(0.005)	−(0.000)
SL-SMOTE	=(0.005)	=(0.352)	=(0.193)	X	=(0.831)	=(0.005)	−(0.000)
ADASYN	=(0.009)	=(0.254)	=(0.130)	=(0.831)	X	−(0.000)	−(0.000)
ICOTE	+(0.000)	+(0.001)	=(0.005)	+(0.000)	+(0.000)	X	=(0.080)
ICOTE + ENN	+(0.000)	+(0.000)	+(0.000)	+(0.000)	+(0.000)	=(0.080)	X

**Table 6:** AUC results of different resampling methods with *k*NN as the base learner.

	None	SMOTE	SMOTE + ENN	SL-SMOTE	ADASYN	ICOTE	ICOTE + ENN
glass1	0.789	0.781	0.776	0.778	0.789	0.869	0.858
ecoli-0_vs_1	0.963	0.970	0.973	0.969	0.963	0.970	0.971
wisconsinimb	0.955	0.965	0.975	0.964	0.957	0.982	0.979
pimaimb	0.664	0.664	0.703	0.692	0.664	0.796	0.832
iris0	1.000	1.000	1.000	1.000	1.000	1.000	1.000
glass0	0.827	0.835	0.832	0.831	0.827	0.913	0.926
yeast1	0.634	0.649	0.671	0.644	0.634	0.849	0.889
habermanimb	0.547	0.582	0.612	0.565	0.547	0.764	0.807
vehicle2	0.940	0.938	0.936	0.938	0.938	0.975	0.983
vehicle1	0.660	0.658	0.709	0.651	0.630	0.884	0.923
vehicle3	0.665	0.677	0.700	0.690	0.676	0.878	0.870
glass-0-1-2-3_vs_4-5-6	0.915	0.949	0.959	0.942	0.912	0.981	0.994
vehicle0	0.911	0.931	0.920	0.925	0.910	0.982	0.986
ecoli1	0.859	0.865	0.881	0.864	0.797	0.942	0.991
new-thyroid2	0.949	0.967	0.961	0.981	0.980	0.992	0.997
new-thyroid1	0.949	0.955	0.946	0.983	0.977	0.992	0.994
ecoli2	0.862	0.931	0.926	0.909	0.906	0.972	0.996
glass6	0.813	0.888	0.874	0.888	0.871	0.992	0.992
yeast3	0.857	0.868	0.866	0.866	0.818	0.973	0.994
yeast-2_vs_4	0.833	0.902	0.877	0.875	0.852	0.981	1.000
yeast-0-5-6-7-9_vs_4	0.680	0.784	0.788	0.757	0.702	0.965	0.988
vowel0	0.971	0.998	0.999	0.996	1.000	1.000	1.000
glass-0-1-6_vs_2	0.619	0.665	0.647	0.684	0.577	0.954	0.976
glass2	0.669	0.690	0.738	0.696	0.601	0.962	0.977
shuttle-c0-vs-c4	1.000	0.996	0.996	0.996	0.996	1.000	1.000
yeast-1_vs_7	0.594	0.713	0.727	0.738	0.646	0.972	0.982
glass4	0.793	0.894	0.915	0.882	0.818	0.980	0.995
ecoli4	0.814	0.961	0.928	0.881	0.870	0.992	0.997
glass-0-1-6_vs_5	0.891	0.874	0.921	0.871	0.836	0.989	0.997
shuttle-c2-vs-c4	1.000	0.996	0.996	1.000	0.950	1.000	1.000
yeast-1-4-5-8_vs_7	0.500	0.628	0.675	0.642	0.574	0.972	0.986
glass5	0.898	0.921	0.921	0.918	0.893	0.990	0.995
yeast-2_vs_8	0.500	0.798	0.816	0.781	0.768	0.985	0.994
yeast4	0.595	0.766	0.797	0.720	0.667	0.918	0.957
yeast-1-2-8-9_vs_7	0.616	0.601	0.622	0.595	0.553	0.975	0.992
yeast5	0.883	0.952	0.938	0.942	0.846	0.992	0.999
ecoli-0-1-3-7_vs_2-6	0.748	0.837	0.832	0.832	0.843	0.987	0.996
yeast6	0.712	0.865	0.856	0.867	0.748	0.987	0.996

**Table 7:** AUC results of different resampling methods with C4.5 as the base learner.

	None	SMOTE	SMOTE + ENN	SL-SMOTE	ADASYN	ICOTE	ICOTE + ENN
glass1	0.733	0.695	0.718	0.726	0.727	0.790	0.783
ecoli-0_vs_1	0.983	0.983	0.983	0.983	0.959	0.975	0.949
wisconsinimb	0.948	0.960	0.940	0.943	0.952	0.967	0.968
pimaimb	0.703	0.725	0.712	0.714	0.705	0.763	0.837
iris0	0.990	0.990	0.990	0.990	0.990	0.990	0.995
glass0	0.817	0.782	0.808	0.756	0.788	0.851	0.871
yeast1	0.668	0.710	0.710	0.719	0.696	0.834	0.829
habermanimb	0.576	0.636	0.649	0.632	0.637	0.758	0.807
vehicle2	0.949	0.959	0.952	0.945	0.939	0.966	0.962
vehicle1	0.660	0.695	0.754	0.733	0.698	0.810	0.869
vehicle3	0.665	0.708	0.716	0.743	0.731	0.845	0.829
glass-0-1-2-3_vs_4-5-6	0.915	0.886	0.916	0.949	0.884	0.957	0.965
vehicle0	0.925	0.936	0.943	0.927	0.939	0.957	0.973
ecoli1	0.859	0.893	0.872	0.901	0.888	0.917	0.951
new-thyroid2	0.949	0.927	0.919	0.966	0.972	0.983	0.986
new-thyroid1	0.949	0.938	0.952	0.930	0.955	0.981	0.977
ecoli2	0.862	0.894	0.877	0.872	0.902	0.940	0.942
glass6	0.813	0.892	0.917	0.863	0.853	0.954	0.975
yeast3	0.857	0.904	0.908	0.906	0.911	0.970	0.983
yeast-2_vs_4	0.833	0.895	0.883	0.859	0.865	0.974	0.980
yeast-0-5-6-7-9_vs_4	0.680	0.830	0.745	0.800	0.725	0.932	0.949
vowel0	0.971	0.979	0.988	0.959	0.969	0.992	0.990
glass-0-1-6_vs_2	0.619	0.712	0.625	0.741	0.580	0.911	0.969
glass2	0.669	0.687	0.756	0.753	0.730	0.929	0.954
shuttle-c0-vs-c4	1.000	0.999	1.000	0.994	0.999	0.999	1.000
yeast-1_vs_7	0.594	0.703	0.657	0.704	0.675	0.930	0.938
glass4	0.793	0.855	0.915	0.816	0.824	0.965	0.979
ecoli4	0.814	0.951	0.923	0.882	0.876	0.987	0.987
glass-0-1-6_vs_5	0.891	0.957	0.919	0.796	0.930	0.994	0.997
shuttle-c2-vs-c4	1.000	0.996	1.000	0.922	0.996	1.000	1.000
yeast-1-4-5-8_vs_7	0.500	0.603	0.587	0.593	0.519	0.931	0.945
glass5	0.898	0.978	0.928	0.846	0.880	0.998	1.000
yeast-2_vs_8	0.500	0.824	0.844	0.814	0.653	0.987	0.985
yeast4	0.595	0.750	0.783	0.780	0.734	0.922	0.923
yeast-1-2-8-9_vs_7	0.616	0.674	0.612	0.517	0.686	0.963	0.959
yeast5	0.883	0.941	0.972	0.947	0.927	0.991	0.995
ecoli-0-1-3-7_vs_2-6	0.748	0.619	0.717	0.717	0.623	0.985	0.989
yeast6	0.712	0.831	0.845	0.811	0.806	0.985	0.984

**Table 8:** AUC results of different resampling methods with SVM as the base learner.

	None	SMOTE	SMOTE + ENN	SL-SMOTE	ADASYN	ICOTE	ICOTE + ENN
glass1	0.496	0.612	0.651	0.612	0.609	0.630	0.570
ecoli-0_vs_1	0.967	0.980	0.983	0.980	0.962	0.982	0.949
wisconsinimb	0.967	0.971	0.970	0.971	0.976	0.970	0.973
pimaimb	0.719	0.742	0.742	0.750	0.747	0.745	0.821
iris0	1.000	1.000	1.000	1.000	1.000	1.000	1.000
glass0	0.691	0.752	0.749	0.734	0.735	0.760	0.806
yeast1	0.573	0.706	0.712	0.702	0.707	0.721	0.764
habermanimb	0.504	0.626	0.645	0.622	0.626	0.636	0.756
vehicle2	0.953	0.950	0.953	0.944	0.950	0.960	0.962
vehicle1	0.720	0.820	0.816	0.796	0.814	0.817	0.887
vehicle3	0.713	0.792	0.803	0.789	0.780	0.790	0.814
glass-0-1-2-3_vs_4-5-6	0.904	0.918	0.916	0.915	0.906	0.923	0.938
vehicle0	0.949	0.959	0.962	0.946	0.959	0.968	0.980
ecoli1	0.819	0.906	0.900	0.906	0.900	0.904	0.921
new-thyroid2	0.983	0.975	0.969	0.972	0.975	0.997	1.000
new-thyroid1	0.983	0.981	0.975	0.978	0.981	0.997	1.000
ecoli2	0.735	0.912	0.912	0.912	0.891	0.905	0.930
glass6	0.920	0.928	0.928	0.909	0.882	0.949	0.950
yeast3	0.630	0.902	0.908	0.904	0.903	0.900	0.927
yeast-2_vs_4	0.669	0.891	0.890	0.885	0.867	0.902	0.918
yeast-0-5-6-7-9_vs_4	0.500	0.774	0.791	0.785	0.786	0.796	0.817
vowel0	0.895	0.968	0.968	0.940	0.963	0.974	0.974
glass-0-1-6_vs_2	0.500	0.593	0.503	0.514	0.536	0.671	0.729
glass2	0.500	0.593	0.593	0.638	0.633	0.703	0.777
shuttle-c0-vs-c4	1.000	0.999	1.000	0.999	0.999	1.000	1.000
yeast-1_vs_7	0.500	0.748	0.753	0.759	0.760	0.766	0.782
glass4	0.559	0.898	0.905	0.918	0.900	0.960	0.969
ecoli4	0.575	0.948	0.935	0.945	0.910	0.979	0.981
glass-0-1-6_vs_5	0.497	0.937	0.943	0.920	0.951	0.969	0.971
shuttle-c2-vs-c4	1.000	0.975	0.996	0.910	0.980	1.000	1.000
yeast-1-4-5-8_vs_7	0.500	0.634	0.627	0.631	0.615	0.689	0.712
glass5	0.500	0.939	0.944	0.922	0.946	0.968	0.967
yeast-2_vs_8	0.774	0.761	0.763	0.742	0.735	0.842	0.830
yeast4	0.500	0.823	0.815	0.821	0.822	0.771	0.763
yeast-1-2-8-9_vs_7	0.500	0.698	0.699	0.720	0.711	0.756	0.753
yeast5	0.500	0.963	0.962	0.960	0.961	0.965	0.975
ecoli-0-1-3-7_vs_2-6	0.850	0.842	0.851	0.822	0.790	0.967	0.970
yeast6	0.500	0.886	0.873	0.887	0.862	0.874	0.883

## Appendix

See Tables 6, 7, and 8.
